# 610. Tele-ID Consult Services at Academic Medical Centers: Experience and Outcomes During the SARS-CoV-2 Pandemic

**DOI:** 10.1093/ofid/ofab466.808

**Published:** 2021-12-04

**Authors:** Aryn M Andrzejewski, Rima Abdel-Massih, Rima Abdel-Massih, John Mellors, Nupur Gupta

**Affiliations:** 1 University of Pittsburgh Medical Center, Pittsburgh, PA; 2 University of Pittsburgh, Pittsburgh, PA

## Abstract

**Background:**

Remote telemedicine ID consults (Tele-ID) appear to be effective for inpatients at community hospitals. Tele-ID is not used at academic medical centers (AMCs) because of the availability of onsite ID physicians. During the COVID-19 pandemic, intra-hospital Tele-ID was implemented because of insufficient PPE and the risk of SARS-CoV-2 exposure. To understand the effectiveness of intra-hospital Tele-ID, we compared outcomes following Tele vs. in-person ID consultation.

**Methods:**

This is a longitudinal, matched, case-control study at two tertiary AMCs in Pittsburgh. Cases were evaluated via Tele-ID only (video, e-consults +/- inpatient phone call) between 3/1/20 – 5/31/20. Controls had in-person consults between 3/1/19 – 11/30/19 matched to cases by sex, race, ethnicity, transplant, age, BMI, Elixhauser score, and ID-specific coded diagnosis. Both groups were evaluated by existing general ID (GID) or transplant ID (TID) service physicians. Patients with COVID-19 diagnosis were excluded. Outcomes included ICU admission, hospital and ICU length of stay (LOS), in-hospital, 30 and 60-day mortality, and 30 and 60-day readmission.

**Results:**

Among the Tele-ID group, 125 inpatients were evaluated by GID and 81 by TID. The majority were Caucasian, male, and non-ICU (Table 1). A broad range of ID diagnosis were made, most commonly bacteremia and pneumonia (Fig 1). Average hospital LOS post-ID consult was 6.26 days (GID) and 6.5 days (TID). For ICU patients, average LOS was 12 days (GID) and 7.6 days (TID). There were 5 (4%) and 3 (3.7%) in-hospital deaths, and 3 (2.4%) and 5 (6.2%) deaths at 30 days for GID and TID, respectively (Table 1). 65 cases could be matched to 633 controls by exact ID coded diagnosis (Table 2). Comparison of Tele-ID cases to in-person controls showed shorter ICU LOS (118.1 vs 269.2 hours; p = 0.002) and lower 30-day readmission (5.1 vs 17.3%; p = 0.004) for cases (Table 2). ICU admission and mortality were similar.

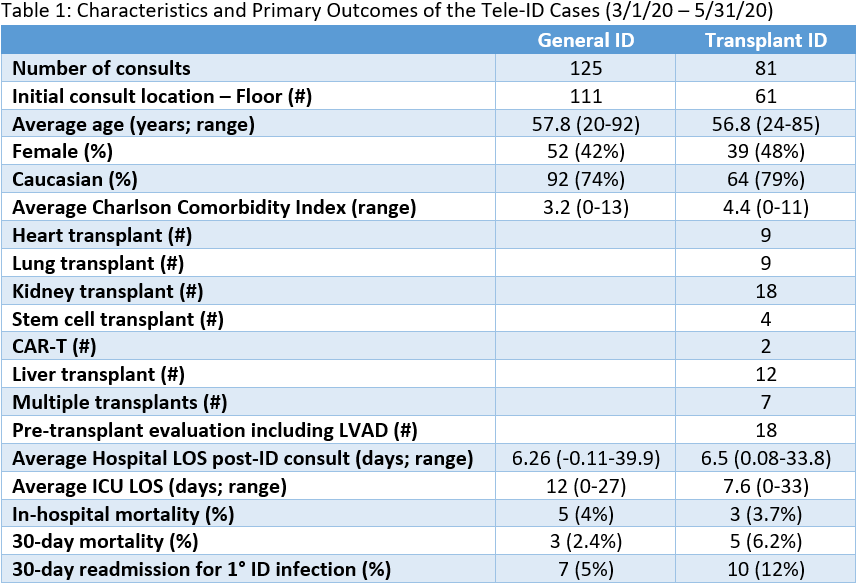

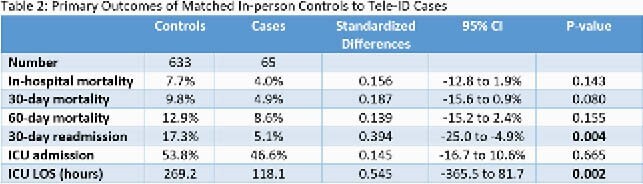

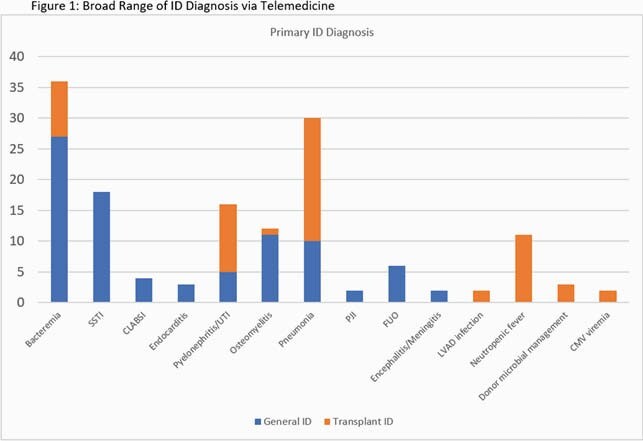

**Conclusion:**

During the pandemic, intra-hospital Tele-ID proved to be an effective alternative to in-person ID consults at large AMCs, as evidenced by shorter ICU LOS and lower 30-day readmission for Tele-ID, and no difference in mortality. This experience suggests that Tele-ID could be used at AMCs as an alternative to in-person consults in non-pandemic settings.

**Disclosures:**

**Rima Abdel-Massih, MD**, **Infectious Disease Connect** (Employee, Director of Clinical Operations) **Rima Abdel-Massih, MD**, Infectious Disease Connect (Individual(s) Involved: Self): Chief Medical Officer, Other Financial or Material Support, Other Financial or Material Support, Shareholder **John Mellors, MD**, **Abound Bio, Inc.** (Shareholder)**Accelevir** (Consultant)**Co-Crystal Pharma, Inc.** (Other Financial or Material Support, Share Options)**Gilead Sciences, Inc.** (Advisor or Review Panel member, Research Grant or Support)**Infectious DIseases Connect** (Other Financial or Material Support, Share Options)**Janssen** (Consultant)**Merck** (Consultant)

